# Coronary steal by left atrial myxoma: a case report

**DOI:** 10.4076/1757-1626-2-6741

**Published:** 2009-06-18

**Authors:** Jose Rubio Alvarez, Juan Sierra Quiroga, Jose Manuel Martinez Cereijo, Laura Reija Lopez

**Affiliations:** Department of Cardiac Surgery, University Hospital of Santiago de Compostela15706, La Choupana SNSpain

## Abstract

This report describes a 41-year-old man who had atypical angina resulting from coronary steal by left atrial myxoma. The tumor was completely excised and the patient was symptoms free after operation.

## Case presentation

**Figure 1.**
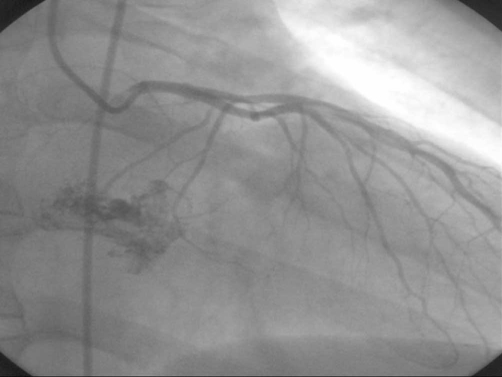
Left coronary angiograms demonstrating vascular supply to the myxoma.

A 41-year-old Caucasian man with one year history of atypical angina and without risks for coronary artery disease , was admitted to the hospital (7-11-2001) presenting an acute coronary syndrome because he had an episode of sudden angina which was accompanied by nausea and sweating . Patient was a non smoker and did not consume alcohol. His past medical history was unremarkable. Emergency coronary angiography showed an abnormal vascular image (Figure 1) evoking an atrial mass vascularized and supplied by the circumflex coronary artery.

**Figure 2.**
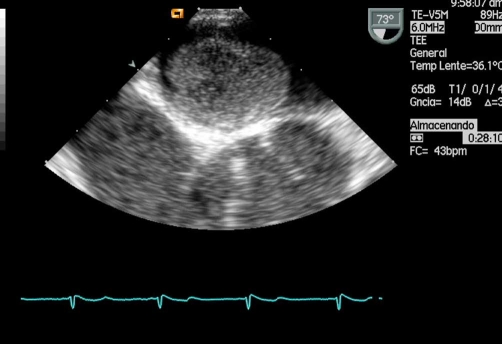
Transthoracic echocardiography shows a large mass near the mitral valve.

The other coronary arteries were normal. The transthoracic echocardiogram showed a mass attached to the interatrial septum and near the mitral valve (Figure 2).

Operative treatment was performed by cardiopulmonary bypass on 8-11-2001. The interatrial septum and the myxoma were removed from the right atrium, the mitral annulus was affected and the mitral valve was replaced with a 27 mm Carbomedics prosthesis (carbomedics inc. Austin TX). A pericardial patch was used to close the atrial septum defect.

**Figure 3.**
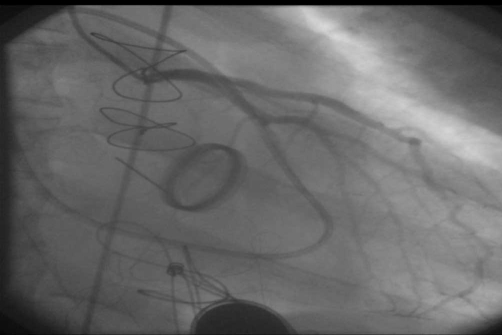
Normal Left coronary angiograms without coronary artery fistula after surgery.

A postoperative coronary angiography which was performed on 13-11-2001 showed a normal coronary tree without coronary artery fistula from the circumflex artery and disappearance of the left atrial mass (Figure 3).

The histological results were compatible with a myxoma.

The patient was symptoms free when last seen in January 2006.

## Discussion

Left atrial myxomas are the most common benign intracardiac tumors and symptoms include dyspnea, chest pain and syncope [1]. In our patient, atypical angina was the only symptom and an episode of sudden angina with a picture of myocardial infarction was the indication for emergency coronary angiography, which showed an atrial mass vascularized and supplied by a fistula from the circumflex coronary artery. Systemic embolization from left atrial myxomas are frequent, however the involvement of coronary artery is extremely rare and atrial myxoma is an unusual cause of myocardial infarction [2].

Operation for an atrial myxoma can be undertaken solely on the base of transthoracic echocardiogram study and this is recommended for follow-up of these patients, especially in familial myxomas where late recurrence has been reported. However, there are authors [3] that recommend that a coronary angiography should be performed preoperatively in all cases.

We do coronary angiography only in patients with angina or in who are at risk for coronary artery disease. Since chest pain is sometimes the only symptom, to perform a coronary angiography is indicates and how in our case the diagnosis can be doing.

With a highly vascularized mass in the left atrium, supplied by the circumflex coronary artery, it is possible to think that the myxoma could explain the onset of myocardial ischemia through a coronary steal effect. This was corroborated in our case by disappearance of the chest pain after surgical excision of the left atrial myxoma.
